# The effects of Task-Oriented Motor Training on gait characteristics of patients with type 2 diabetes neuropathy

**DOI:** 10.1186/s40200-016-0236-8

**Published:** 2016-05-25

**Authors:** Hoda Salsabili, Farid Bahrpeyma, Ali Esteki

**Affiliations:** 1 Department of Physiotherapy, Faculty of Medical Sciences, Tarbiat Modares University, Tehran, Iran; 2 Medical Physics and Engineering Department, Shahid Beheshti University of Medical Science, Tehran, Iran

**Keywords:** Gait training, Type 2 Diabetes, Diabetic Neuropathy, Task-Oriented, Diabetes Rehabilitation

## Abstract

**Background:**

It is known that general gait training improves lower extremity muscle strength and endurance in Diabetes Neuropathy (DN). But, it is still unknown whether Task-Oriented (TO) gait training would change gait biomechanics and the risk of falling in DN. TO gait training focuses on promoting timing and coordination of lower extremity movements through goal-directed practices with sufficient repetition.

**Methods:**

A group of 14 patients with DN participated in a time-series study. All subjects participated in four sessions of assessments (Initial, Pre, Post and Follow-Up). Training was twice a week for 12 weeks. Vertical and horizontal Ground Reaction Forces (GRF), Time Get up and Go (TGUG) and Fall Efficacy Scale-International (FES-I) were evaluated. Gait training started with stepping patterns that progressively changed to complicated patterns of walking. Then, training continued combining walking patterns with upper extremity activities and then ended with treadmill-paced practice.

**Results:**

DN patients significantly increased Second Vertical Peak Force and Horizontal Propulsive Force in addition decrease in Minimum Vertical Force. TGUG significantly decreased while FES-I reflected significant increase after gait training.

**Discussion:**

Conclusively, training not only improved gait performance, confidence in daily activities and attenuated risk of falling, but also helped DN patients to improve feet biomechanics, muscles timing and coordination.

**Conclusions:**

Gait training with respect to principles of motor learning allowed patients to effectively improve through sessions.

## Background

Difficulties in walking of Diabetes Neuropathy (DN) patients results in higher risk of falling and injuries [1]. Falling has been shown to happen as a consequence of gait abnormalities in DN populations [2–5]. Some studies have reported common changes in gait parameters in DN patients including higher gait variability [6], longer stance phase [7] and slower speed of walking [8]. Kinetic parameters also reported some changes such as modified Ground Reaction Forces (GRF) and moments of forces. In the study by Katoulis et al., (1997), the maximum value of the vertical component of the GRF and anterior-posterior (AP) forces was found to be lower in the DN group than control groups [9]. Despite changes in the GRF, Breaking Time was also longer and Center of Pressure overshoots were larger in the diabetic subjects than healthy people [10]. The association between falling and abnormal gait was also demonstrated in diabetes populations [11]. Therefore, understanding mechanisms of changes in the gait parameters has facilitated understandings about fall risk prediction in the DN population [1]. Also, slower walking has been demonstrated as a pro-active strategy to improve walking stability [12]. Altogether, modified gait parameters support that changes in the walking strategy of diabetic patients with peripheral neuropathy have occurred.

Various factors cause gait abnormalities in DN patients. Abnormal muscle performance and modified ankle mobility alter foot biomechanics and lead to abnormal foot loading [13]. Substitution of the vestibular system to control body orientation also has some influences on the control of stability and balance during gait [14].

To improve balance and stability during walking, different type of training approaches have been suggested for DN patients. Allet et al., (2010) recommended gait and balance exercises with function-oriented strengthening [15] and then in the Follow-Up study suggested these specific training exercises on the challenging environment [16]. Lower extremity strength and balance training was also designed as an intervention exercise regimen to improve walking ability of DN patients [17]. In a complementary physiotherapy intervention, treatment on foot roll-over during gait, range of motion, muscle strength of foot and ankle and balance confidence were carried out to improve walking performance of DN patients. Patients showed improvements in the function of dorsiflexion and earlier lateral foot contact with respect to medial forefoot [18]. Similar investigation showed the positive effects of strengthening, stretching and functional training on foot biomechanics of DN patients [19]. In general, most investigations on gait rehabilitation of DN patients focused on strengthening of muscles, balance training and enhancement of gait biomechanics to relieve impairing effects of neuropathy.

Nevertheless, a new line of investigation started to pursue motor deficit rehabilitation with the inspiration of motor learning principles. Recently, VanSwearingen et al. (2011 and 2009) in two studies on older adults included pillars of motor learning to improve gait mechanics. In their study, Task-Oriented (TO) motor learning exercise intervention has been preferred to Impairment-Oriented (IO) exercise intervention in older adults to improve gait performance. In the IO exercises, focus was on the current standard physical therapy for retraining gait and balance by stretching exercises, balance, endurance and strength training. On the other hand, TO intervention is based on principles of motor learning to enhance skilled and smooth control of movements during walking [20, 21]. TO training in gait rehabilitation specifically concentrates on the improvements in gait performance through goal-directed practices with sufficient repetition [22]. However, previous studies on DN patients` gait training did not pay attention to the promotion of gait patterns timing and coordination with respect to motor learning principles.

In the present study, we aimed to recruit TO motor training to determine influences on the gait characteristics of DN patients as an alternative to the traditional gait training. TO motor training was modified based on the DN patients` context of disabilities to encourage the patients to attend repetitive and gait-specific practices. In other words, practices were specifically designed regarding the requirements of smooth walking [23, 24] and designed to have adequate repetition for adopting normal walking by DN patients [25]. Therefore, we implemented various stepping and walking patterns to promote the timing and coordination of the movements in the gait cycles. We planned to focus on recruiting different ankle muscle groups affected by neuropathy, activating groups of lower extremity muscles in complex patterns of walking and then combining complicated patterns of walking with upper extremity exercises. At the end of the training pattern, treadmill training was added to improve speed of walking. Progression toward more complicated and paced patterns of walking was recruited to increase the speed and accuracy of gait performance [26, 27]. Additionally, principles of motor learning including specificity and repetition of practices were followed in the gait training [28]. Therefore, it was hypothesized that TO gait training of DN patients would improve gait biomechanics and function.

## Methods

### Participants

A time-series pre and post-test design was used in this study. A group of 14 patients with diabetic neuropathy was recruited. Neuropathy was detected using Michigan Neuropathy assessment. All subjects were screened to satisfy Michigan Diabetes Neuropathy Score (MDNS). It is a 15-item assessment and a higher score reflects more extensive neuropathy (MDNS > =7) [29, 30]. Moreover, patients had to finish the Time Get Up and Go test (TGUG) in less than 13.5 s as a scale of walking performance to show their independent walking ability [31, 32]. All subjects had controlled blood glucose level by the screening by Glycated Haemoglobin test (9 % > HbA1c > 6.5 %) [33]. Patients were excluded from this study if they had retinopathy, scares under their feet, hypo or hypertension, autonomic neuropathy or any orthopedic and neurologic disorders that influence walking performance. Patients had no experience with similar gait training or balance practices during the last 12 months. No patients changed, nor discontinued their medical prescriptions due to gait training in this study. This study was performed in accordance with the declaration of Helsinki, and it was approved by the Tarbiat Modares Institutional ethical review board (Reference Number: 1789965214). All participants signed informed consent before the start of the study.

### Apparatus and procedures

All patients participated in four sessions of assessments (Initial-evaluation, Pre-training, Post-training, and Follow-Up). The first three sessions of assessments consecutively repeated every 12 weeks, and the Follow-Up session was just 3 weeks after the Post-training session. In the first session, descriptive and demographic information of patients were recorded (Age, Sex, Height, Weight, History of diabetes, HbA1c and Michigan Neuropathy score). In all sessions, patients were subjected to gait kinetic parameters evaluations on force platform, Fall efficiency scale-International (FES-I) and TGUG test. Gait training occurred twice per week, for 12 weeks between Pre-training and Post-training sessions (Training period). Participants did not receive gait training in the weeks between Initial-evaluation and Pre-training (Control period) and between Post-training and Follow-Up sessions (Follow-Up period).

#### Force platform assessment

Force platform (Kistler 9286BA; Winterthur, Switzerland) is used for evaluating kinetic measurements. It was inserted in the middle of the wooden walkway. DN patients were instructed to walk barefoot with preferred speed on the walkway without looking down at the platform. Every patient was familiarized to gait assessment on force platform before the start of data collection. All assessments and gait training were conducted by physical therapist experienced in diabetes neuropathy rehabilitation. Due to the nature of the study, neither the physical therapist, nor patients were blinded to the goals of the study. Three successful full contact foot trials were acquired for the right leg. Sampling rate was 100Hz for recording GRF signal.

All data were processed and variables were calculated using Matlab 2013 (MathWorks Inc., Natick, MA). All GRF signals were filtered with zero lag fourth order low-pass Butterworth filter with cut off frequency at 10 Hz. The Vertical and Horizontal GRF were collected and then normalized to the subject`s weight for standardization of stance phase. The Vertical GRF variables consist of the First and Second Vertical Peak Force, and the Minimum Vertical Force between the two maximum forces [5]. The horizontal GRF variables consisted of the Breaking Force and the Propulsive Force components. Both Propulsive and Breaking Time variables normalized to 100 % of the stance phase. Every GRF variable was averaged over three trials for statistical analyses. Recorded Propulsive Time and Breaking Time variables consist of the time to reach the first peak and the time to return to the ground after the second peak force, respectively [34].

#### Fall Efficiency Scale-International (FES-I)

It is a self-reported 16-item scale of perceived confidence to complete physical daily activities. It is administered to reflect the level of concern about falling in activities inside and outside the home. Each Item score ranges from 1 to 4 for no confidence to complete performing daily activities. Possible total score is 64 in the worst case and 16 in the best condition to do all tasks without any concern of falling [35]. We used the Persian-language version of the FES-I questionnaire to assess fear of falling among DN patients [36]. In the study by Allet et al., (2010), fall efficacy scale was used for diabetic patients after specific gait training in different environments. Compared to the control group, intervention group showed significant improvement in fear of falling [16]. Some studies have been supported Validity of the FES-I questionnaire [35, 37].

#### Time Get Up and Go (TGUG)

Time Get Up and Go is for observing patient and time them while raising from an armchair, walking 3 m, turning, walking back and siting down at the end. This functional mobility scale appears to predict patient`s ability to go outside the home alone [38]. Reliability and Validity of TGUG test for quantifying functional mobility were approved for identifying gait performance [32].

### Intervention and training

Each training session lasted 45 min and subjects were free to take a rest for 2 to 5 min between tasks. The interventions were twice a week for 12 weeks. This program of training was based on the principles of TO motor learning. TO gait training involved different stepping and walking patterns to promote timing and coordination of movements to gain better balance and performance during gait [20, 21]. To have progressions in the difficulty of practices, subjects were instructed to increase the speed of their walking and try to keep themselves accurately on the assigned pathways as much as possible. In other words, participants had to reduce their base of support while performing practices. A Practice session started with stretching exercises to warm up the patient and increase joint range of motions and then moved on to have different stepping patterns. Diverse stepping patterns were designed to recruit different damaged muscle groups including ankle dorsi-flexors and plantar-flexors in the neuropathic patients. Progression involved increasing speed and accuracy of performance while practices became harder in the context of destabilizing subjects with a more difficult pattern of walking. Complicated patterns of walking made patients implement more sensory recourses to provide enough information for controlling balance during walking.

In the next step, complicated patterns of walking were combined with perturbations imposed on the upper extremity. In the upper and lower extremity combined practices, patients had to finish practices with higher attentional demand of controlling objects in their arms, while controlling their walking to be precisely on the walkway. At the end of each session, to promote regular timing of the gait, treadmill-paced training was incorporated with the alternative changes in the assigned speed of movement to regulate coordinated muscular patterns practiced in every session. DN Patients had to start walking with preferred speed and after a while, speed was increased by 10 % and then returned to the comfortable walking. Treadmill training encouraged DN patients to have consistency of walking speed and timing control (Table 1).

**Table 1:** All charactristics of Task-Oriented motor gait training. The table is describing the pattern of practices, a brief descrption of each practice and the specific time for each practice

Practices and patterns		Description of practices	Minutes(Min)
Flexibility		Stretching of lower extremity muscles Hamstrings, Quadriceps, Trunk and Ankle plantar/dorsi flexors	5
Stepping		Stepping instructed on a straight line	
1- Dorsi-stepping 2- Plantar-stepping 3- Forward-Backward stepping		Stepping on the heels with Dorsi-flexion Stepping on the heels with Dorsi-flexion Stepping forward and then backward	2 2 3
4- Cross-stepping		crossing the pathway in stepping	3
5- Tandem-stepping		back to back stepping	3
Simple Walking		Walking instructed on special pathways	
1- Oval 2- Spiral 3- Eight like		Walking on an oval shaped pathway Walking spiral pathway in between obstacles Walking around an eight-shaped pathway	2 2 2
Complicated walking		Walking practices with upper extremity practices	
1- Two-handed ball carrying	Oval Spiral Eight	Walking on an oval shaped pathway with carrying a ball Walking spiral pathway in between obstacles with carrying a ball Walking around an eight-shaped pathway with carrying a ball	1 1 1
2- Throwing ball to the therapist	Oval Spiral Eight	Walking on an oval shaped pathway while throwing back and for the ball to the therapist Walking spiral pathway in between obstacles while throwing back and for the ball to the therapist Walking around an eight-shaped pathway while throwing back and for the ball to the therapist	1 1 1
3- Bouncing ball on the floor	Oval Spiral Eight	Walking on an oval shaped pathway while bouncing a ball on the floor Walking spiral pathway in between obstacles while bouncing a ball on the floor Walking around an eight-shaped pathway while bouncing a ball on the floor	1 1 1
Treadmill paced walking		Alternatively the speed of walking changed	(3 times)
1- Preferred speed 2- 10 % increased speed 3- Preferred speed		Walking with usual speed 10 % increased speed to the usual speed Walking with usual speed	1 2 1
			45 min

### Statistical analysis

Statistical tests included repeated measure analysis of variance (ANOVA) to test mean differences in four sessions of assessments. GRF variables, TGUG and FES-I were inserted in the statistical analyses. We added a Bonferroni corrected model to analyze main effects of training compared to other sessions. SPSS statistics software, version 21 (SPSS Inc.; Chicago, Illinois) was used for all statistical analyses. Significant level of differences was set to be 0.05 for all analyses.

## Results

Fourteen DN patients (eight females and six males) participated in the study (56.5 ± 7.057 years). Demographic data of included patients are reported in Table 2.

**Table 2:** DN Patients’ (*N* = 14) baseline descriptive and demographic characteristics

Descriptive Information	Patients
Age	56.5 ± 7.05
Sex	eight females and six males
Height	165.28 ± 7.69 Cm
Weight	80.92 ± 10.99 Kg
History of Diabetes	12.57 ± 5.59 Years
Michigan Neuropathy score	15.6 ± 8.60
HbA1c	7.35 ± 1.03

### Ground Reaction Force (GRF)

After gait training, repeated measure ANOVA showed significant differences in the Minimum Vertical Force and in the Second Vertical Peak Force (F_3,39_ = 4.095, *P* = 0.011 and F_3,39_ = 2.943, *P* = 0.041). First Peak Vertical Force, Breaking Time and Propulsive Time did not show any significant differences between sessions of assessments (*P* > 0.05). Horizontal GRF, Propulsive and Breaking Forces showed significant differences in the repeated measure ANOVA within subject effects, respectively (F_3,39_ = 6.462, *P* = 0.001 and F_3,39_ = 3.606, *P* = 0.019).

In the Bonferroni analysis, Minimum Vertical Force demonstrated significantly lower forces in the mid-foot contact with the ground in DN patients following gait training in the Post-training session than Pre-training session (*P* = 0.007). Meanwhile, Minimum Vertical Force was not significantly different in the Follow-Up session from Pre-training session (*P* = 0.085).

Additionally, Second Vertical Peak Force reported significantly higher during gait push-off in post- training than Pre-training session (*P* = 0.034). They did not display the effects of training in the Follow-Up sessions (*P* > 0.05).

Pairwise comparisons of Horizontal GRF, and Propulsive Force reflected significantly higher forces in the Post-training and Follow-Up sessions than Pre-training (*P* = 0.006 and *P* = 0.009), respectively. Breaking Force in the pairwise comparison, though we found significant differences in the main effects, did not show any significant differences between sessions (*P* > 0.05). Also, Minimum Vertical Force, Propulsive Force and Second Vertical Peak Force did not indicate any significant differences in pairwise comparison between Pre-training and Initial-evaluation sessions, and between Post-training and Follow-Up sessions (*P* > 0.05).

**Fig. 1:**
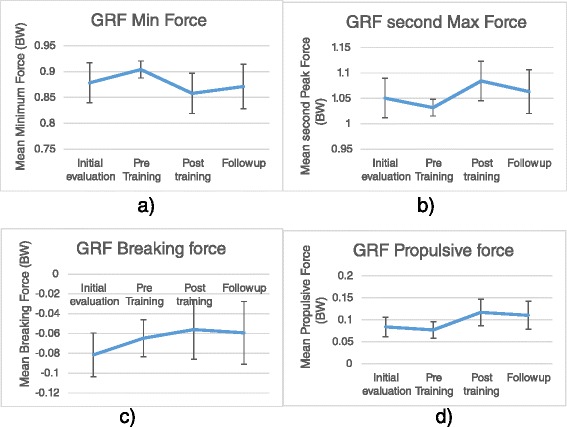
Mean of GRF in Initial-evaluation, Pre-training, Post-training, Follow-Up sessions of assessments. **a** Vertical GRF Min Force, **b**) Vertical GRF second peak force, **c**) Horizontal GRF Breaking force and **d**) Horizontal GRF Propulsive Force. All GRF curves normalized to each participant`s body weight in the DN group

DN patients demonstrated better performance in foot clearance by the help of higher forces in Second Vertical Peak and Propulsive Forces during foot push-off and depicted lower Min Vertical force during mid-foot contact with the ground (Fig. 1).

### Fall efficacy scale-International

In the analyses of Gait Efficiency scale, repeated measure ANOVA reflected significant differences in the within-subjects effects of the scores (F_3,39_ = 7.036, *P* < 0.05). Among different assessments, Fall efficiency scores indicated better confidence of participants in the Post-training and Follow-Up sessions than Pre-training session (*P* = 0.008, *P* = 0.014). There were no significant differences between Initial-evaluation and Pre-training session (*P* > 0.05) or between Post-training and Follow-Up sessions (*P* > 0.05). Subject`s confidence in their ability showed a 9.5 point decrease to display lower fear of falling with gait training in the activities required for controlling balance.

**Fig. 2:**
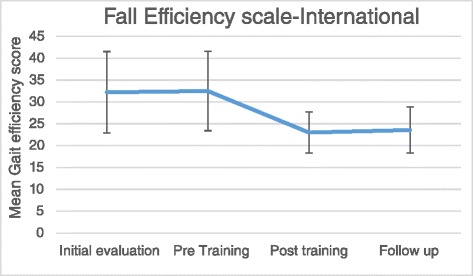
Mean of fall efficacy scale score through assessments

FES-I indicated higher confidence of participants following gait training and lower fear of falling in daily activities in the Post-training and Follow-Up sessions (Fig. 2).

### Time Get Up and Go (TGUG)

**Fig. 3:**
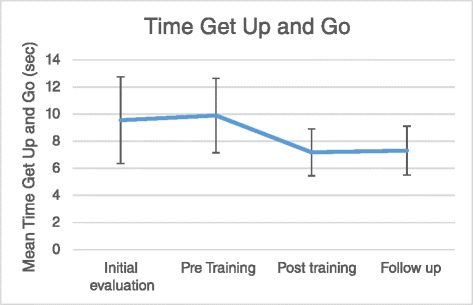
Mean of time TGUG test scores through sessions of assessments

In the functional examination of walking by TGUG, repeated measure ANOVA showed significantly different results in the within subject effects (F_3,39_ = 4.845, *P* = 0.005). In the pairwise comparisons, participants indicated significantly faster completion of TGUG in the Post-training and Follow-Up sessions than the Pre-training session (*P* = 0.03 and *P* = 0.043). They failed to reach any significant differences between the Initial-evaluation and Pre-training assessments (*P* = 0.982) and Post-training and Follow-Up sessions (*P* = 0.999). Figure 3 shows the trend of changes in the TGUG task completion by subjects through four sessions of assessments.

## Discussion

In the present study we examined the effects of TO motor gait training on biomechanical and functional DN patients’ responses. TO motor gait training led to gain better biomechanical and functional responses during walking in DN patients. TO motor gait training intervention had a target to correct deficits in the muscle patterns of stepping and integrating lower extremity mechanics with the phases of the gait [21]. This therapeutic approach of walking in DN patients resulted in higher Second Vertical Peak GRF, lower Minimum Vertical GRF, higher Propulsive Force, less time to finish TGUG and lower scores in FES-I. Generally, these improvements also appeared to promote gait efficiency and better confidence to activities during gait.

Regarding the hypothesis of the study, increased Second Vertical Peak Force and Propulsive Force in the analyses of the GRF in addition to the reduction of the minimum peak force showed how DN patients changed these forces that have already been under the impact of neuropathy. In fact, DN patients had better foot clearance with stronger push-off on the floor after training and spent less forces in the mid stance. TO motor gait training helped DN patients to have better neural-mechanical adaptation in order to enhance mechanical stresses required for foot clearance and reduce forces during roll over the foot.

In the study by Katoulis et al. (1997), maximum value of the vertical component of GRF was found to be higher in the control groups than DN patients. Also, they found the max anterior-posterior forces was higher in the diabetes group than DN patients with previous plantar ulcers. They attributed differences to higher speed of walking in the control group than the diabetic and neuropathic groups. The authors claimed that slower walking speed is due to decreased plantar flexor muscle strength and ankle mobility in the diabetic group. These diminished abilities in the DN patients inhibit generating enough plantar flexors moment and power to push off during terminal stance [4, 5]. In our study, compared to the Katoulis et al., (1997), we found no significant differences in the Breaking and Propulsive time in the gait cycle. However, the result is showing higher timely coordinated activation of plantar flexor groups of ankle muscles in the push-off and mid-stance.

Consistently, in the study by Shaw et al., (1998), neuropathy was claimed to mostly associate with the change in the time pattern of forces transmitted through the foot. In the neuropathic patients, the shift of the timing increased heel forces [39]. In our study, we found no significant changes in the heel transmitted force while force transmitted by metatarsals increased possibly due to higher activation of plantar muscles in terminal stance rather than increasing speed of gait in DN patients.

As the first explanation, TO gait training increase proprioceptive inputs causing better body progression on feet. The sensory burdens on the neuromuscular system will make it generate responses according to the afferent information caused by mechanical loads placed on the foot [18]. In DN patients, destruction of proprioception system made DN patients susceptible to more falling [40].

Another possible reason to explain observed changes is relevant to what has been discussed in the studies by Mueller et al., (1994 and 1995). They have reported during neuropathic gait, strength of ankle plantar flexors will reduce and the body adopt hip muscle groups for walking whereby the leg is pulled forward from the hip rather than being pushed forward by the feet [4, 41]. Thereby, DN patients switch from ankle to hip muscle groups [42, 43]. Relevantly, our findings can support that DN patients switched to better recruitment of ankle muscles in push-off. In this study, the stepping part of training was concentrated on the exercises which made a high level of activations in ankle muscles and specifically in different muscle groups. TO gait training helped recruiting different groups of ankle muscles and reinforced them to work together and coordinate in timing to get better foot roll-over and push-off [44].

Apparently, the principles that were borrowed form motor learning were lending support to the effects of TO gait training. In every practice session, there was enough repetition as the first considered motor learning principle. Repetition allowed DN patients to make adjustment to their walking with the assigned path of practicing on the floor. Studies on the effect of repetition of practice has been proved underlying role of practicing in facilitating coordination and control of muscles [45].

Secondly, DN patients had to represent higher external focus of attention to the accuracy of their stepping on the walking pathway. Training accuracy made patients pay special attention to decrease their base of support. Previous investigation has revealed diabetes patients showed wider base of support during walking [46]. In the present study, DN patients had to tolerate destabilizing effects of small base of support by paying more attention to instructions of the tasks and in return exert better controlling over their balance during practices. In support of this idea, it has been confirmed that incorporation into external focus of attention in the rehabilitation practices would potentially enhance the efficacy of a training program [28, 47].

Similarly, specificity of practice as the third pillar of the training explains better mechanics and performance of gait after training. The emphasized tasks in the interventions were specifically focused on different patterns of walking. Normally, specific training of any task results in the improvements of the trainees in similar tasks and conditions. In other words, our intervention was goal-directed to improve walking performance and control [23, 28].

Consistently, in further support some studies showed the benefits of TO motor gait training. Van Swearington et al., (2014 and 2012) improved timing and coordination of walking in older adults and showed a preference of TO gait training to impairment based training [20, 21]. Moreover, Salsabili et al., (2011 and 2013), reflected the effectiveness of context-specific balance training in improving postural control in DN patients [48, 49]. Van Peppen et al., (2004) also reviewed the impact of task-oriented physical therapy exercises on the functional outcome of stroke patients. It was described in this study that the TO approach to training effectively improved mobility related activities [50]. Drabsch et al., (1998) also suggested the assistive effects of TO training to improve performance in individuals with total hip replacement [51].

In the present study, we have built TO trainings in connection with motor intervention. Purposefully, it was targeted to correct deficits in the stepping patterns and walking specific to the gait phases. Given that the structure of the TO gait training program was built on the motor learning principals, some effects on the biomechanical behavior of the feet during walking was presumed. Additional to the findings in the GRF modifications, DN patients also showed functionally better performance in the TGUG and fall efficacy scale. Relevantly, diabetes patients fear of falling base on the FES-I showed higher concern in controlling their balance in daily activities [52, 53]. Thus, TO gait trainings successfully reduced fear of falling and allowed DN patients to be more confident to their daily activities.

Additionally, the cut-off score for the TGUG assessment was revealed to be 10.7 to show diabetes patients at risk of falling [54]. In this study, we found lower scores of DN patients after gait training which is supporting improvement in the control of balance in DN patients. The average TGUG score was 9.89 s Pre-training and it was reduced to 7.18 s in the Post-training session. We found 2.71 s, or 27 % decline in the time to finish TGUG test. In one recent study, minimal detectable change was 2 s for Parkinson patients in TGUG tests [55]. This study also confirms that DN patients gained significant improvements after TO gait training. Moreover, based on the study by Shumway et al., (2000), the mean time to finish TGUG test is 8 s for an aged-matched community-dwelling adults to our study. Therefore, DN patients generally finished TGUG test similarly to healthy subjects and revealed lower risk of falling after TO gait training.

There were some limitations to the present study, including lack of age-matched healthy subjects in this study. Although all DN patients benefit from the privileges of TO gait training, but a group of healthy subjects would help to recognize the trends of changes more effectively. Additionally, the experienced physical therapist was not blinded to the effects of TO gait training. Thus, this might inspired subjects to better perform Post-training assessments. We need also further analysis to report electrical activity of ankle muscles to know the exact timing of activating ankle and knee muscles to support the results of this study.

## Conclusions

In general, TO motor gait training for DN patients not only enhanced performance during walking, but also modified and improved foot mechanics during walking. Changes in the provided sensorimotor information and enhanced muscle abilities can be regarded as reliable contributions for gait responses in DN patients. Conclusively, gait training with respect to principles of motor learning allowed patients to effectively improve through sessions.

## Abbreviations

DN: Diabetes Neuropathy
FES-I: Fall efficiency scale-International
GRF: Ground Reaction Forces
IO: Impairment-Oriented
TGUG: Time Get Up and Go test
TO: Task-Oriented
